# Signaling Pathways in Pregnancy

**DOI:** 10.3390/cells11091385

**Published:** 2022-04-20

**Authors:** Giovanni Tossetta, Daniela Marzioni

**Affiliations:** 1 Department of Experimental and Clinical Medicine, Università Politecnica delle Marche, 60126 Ancona, Italy; d.marzioni@univpm.it; 2 Clinic of Obstetrics and Gynaecology, Department of Clinical Sciences, Salesi Hospital, Azienda Ospedaliero Universitaria, 60126 Ancona, Italy

We are pleased to present this Special Issue of *Cells*, entitled ‘Signaling Pathways in Pregnancy’. The placenta is an essential fetal organ which facilitates the development, growth and protection of human and mammal fetuses, playing different and fundamental functions during pregnancy. Placental villous tree development and differentiation are tightly regulated during pregnancy by a great number of growth factors, their receptors and other types of molecules that regulate placental cell proliferation, differentiation, migration and invasion. Achieving the correct balance of these processes by the activation of different pathways regulating the expression of certain genes is critical for a successful pregnancy. Alteration in placental development is associated with pregnancy pathologies, including preeclampsia (PE) [1], fetal growth restriction (FGR) [2], gestational trophoblastic diseases (GTD) [3], preterm delivery [4] and gestational diabetes mellitus (GDM) [5]. In addition, gestation can also be compromised by the exposure to exogenous agents such as bacteria [6,7], viruses [8], chemicals and natural compounds [9,10] that can also affect placental development and function by altering important signaling pathways. Many of the disorders/pathologies associated to placental alteration result in increased maternal and fetal mortality and morbidity and can lead to life-long health complications for both mother and child. 

It has been reported that many signaling pathways are altered in pregnancy complications (such as PE and GDM), including the Wnt/β-catenin, TGFβ/SMAD, PI3K/AKT/mTOR and JAK/STAT pathways [11,12,13,14]. In addition, viral and bacterial infection causes an increase in inflammatory cytokines that can impair the normal function of placenta and amniotic membranes [7,8,15]. Moreover, oxidative stress and inflammation found in these pregnancy complications [4,6,9,11,14] can further worsen these pathologies, leading to systemic endothelial dysfunction [16,17].

The availability of biomarkers that allow an early (some of them already in the first trimester [18,19,20]) diagnosis of many of the above-mentioned pregnancy complications would make it possible to carry out an early treatment of the pathology in order to improve the outcome of the pregnancy or resolve the disease. As reported in literature, many natural and synthetic compounds that act in activating or inhibiting many impaired signaling in pathological pregnancies could be used to treat these patients. In particular, the major commonly used compounds are low-molecular-weight heparin and salicylic acid. In addition, other compounds including curcumin and resveratrol could be used as supplements to ameliorate the disease [21,22,23,24].

Understanding the mechanisms involved in the regulation of human placenta development and the main modulators involved in the activation/inhibition of the signaling pathways altered in pathological conditions can help to open a new prospective in the treatment of these pregnancy pathologies. Thus, the aim of this Special Issue is to provide an overview of the signaling pathways involved in placental development in normal and pathological conditions.
